# Whole Genome Sequencing of Four Representatives From the Admixed Population of the United Arab Emirates

**DOI:** 10.3389/fgene.2020.00681

**Published:** 2020-07-09

**Authors:** Gihan Daw Elbait, Andreas Henschel, Guan K. Tay, Habiba S. Al Safar

**Affiliations:** ^1^Center for Biotechnology, Khalifa University of Science and Technology, Abu Dhabi, United Arab Emirates; ^2^Department of Computer Science, Khalifa University of Science and Technology, Abu Dhabi, United Arab Emirates; ^3^Department of Biomedical Engineering, Khalifa University of Science and Technology, Abu Dhabi, United Arab Emirates; ^4^Division of Psychiatry, Faculty of Health and Medical Sciences, The University of Western Australia, Crawley, WA, Australia; ^5^School of Medical and Health Sciences, Edith Cowan University, Joondalup, WA, Australia; ^6^Department of Genetics and Molecular Biology, Collage of Medicine and Health Sciences, Khalifa University of Science and Technology, Abu Dhabi, United Arab Emirates

**Keywords:** whole genome sequencing, next generation sequence analysis, population admixture, United Arab Emirates, population-specific allele frequencies

## Abstract

Whole genome sequences (WGS) of four nationals of the United Arab Emirates (UAE) at an average coverage of 33X have been completed and described. The selection of suitable subpopulation representatives was informed by a preceding comprehensive population structure analysis. Representatives were chosen based on their central location within the subpopulation on a principal component analysis (PCA) and the degree to which they were admixed. Novel genomic variations among the different subgroups of the UAE population are reported here. Specifically, the WGS analysis identified 4,161,067–4,798,806 variants in the four individual samples, where approximately 80% were single nucleotide polymorphisms (SNPs) and 20% were insertions or deletions (indels). An average of 2.75% was found to be novel variants according to dbSNP (build 151). This is the first report of structural variants (SV) from WGS data from UAE nationals. There were 15,677–20,339 called SVs, of which around 13.5% were novel. The four samples shared 1,399,178 variants, each with distinct variants as follows: 1,085,524 (for the individual denoted as UAE S011), 1,228,559 (UAE S012), 791,072 (UAE S013), and 906,818 (UAE S014). These results show a previously unappreciated population diversity in the region. The synergy of WGS and genotype array data was demonstrated through variant annotation of the former using 2.3 million allele frequencies for the local population derived from the latter technology platform. This novel approach of combining breadth and depth of array and WGS technologies has guided the choice of population genetic representatives and provides complementary, regionalized allele frequency annotation to new genomes comprising millions of loci.

## Introduction

Comprehensive knowledge of genetic variation for entire populations is essential to improve our understanding of the medical relevance and for functional interpretation of DNA sequence changes. Since the inception of the 1,000 Genomes project (Genomes Project Consortium et al., 2010), a deluge of genome sequencing projects (Bentley et al., 2008; Kim et al., 2009; Fujimoto et al., 2010; Alabdulkareem et al., 2015; John et al., 2015; Thareja et al., 2015; Almal et al., 2019; AlSafar et al., 2019) have been initiated to identify the genetic variations among different human subpopulations.

Despite the distinctive population structure and ethnic diversity of the Arabian Peninsula including the variations seen in the United Arab Emirates (UAE), genomic representation of middle-eastern populations in the global genome catalog remain underrepresented with only 0.08% of human genetic sequences in current public databases being from populations of the region (Popejoy and Fullerton, 2016).

The geographical location of the Arabian Peninsula at the crossroads of Africa, Europe, and Asia has enabled the bidirectional transcontinental gene flow into and out of the region throughout human history (Omberg et al., 2012). This area is endowed with a wealth of history, cultural heritage and human ethnic diversity (Teebi, 2010). Therefore, the study of the Arabian Peninsula would provide an opportunity to better understand early to modern changes in human demographic patterns through selection, admixture, and gene flow that has been driven by migration out of and back into Africa.

The state of UAE, located at the south eastern corner of the Arabian Peninsula stretches along the coastline of the Arabian Gulf on the north, is surrounded by Oman, Saudi Arabia, Qatar, and Iran. Although its pivotal geographic position and diverse population makes it important to study in order to understand the patterns of migration in the region, the ancestry of its contemporary population remains obscure as there has been no in-depth genetic study presented to date except for two recent publications (Fernandes et al., 2019; Tay et al., 2020).

In addition to the detailed early migration movements in the Arabian Peninsula described in Tay et al. (2020), the area has experienced relatively recent waves of population traffic. The territory bounded by the borders of the UAE has undergone a seasonal influx of Arab tribes from Yemen as well as Central and Northern Arabia (Bey, 1996). Moreover, some of the traditional nomadic populations roamed the region and took possession of economically viable locations of this land (Tadmouri et al., 1987). Intermixing occurred with the people of Central/South Asia, a group collectively referred to as Persians, influenced by the influx of traders, scholars, and philosophers, as well as immigrants along both the coastlines of Asia and Arabian Peninsula on either side of the Arabian Gulf (Tadmouri et al., 1987).

Regional efforts in Qatar (Omberg et al., 2012; Fakhro et al., 2016) and Kuwait (Alsmadi et al., 2014; John et al., 2015; Thareja et al., 2015) have provided an initial genetic perspective but there are arguably differences in ethnic composition arising from differences in geographical location and tribal structures, which warrants a thorough population stratification analysis of the UAE. Recently, the first whole genome sequences (WGS) of Emirati nationals was reported in AlSafar et al. (2019) from two samples both with mainly Middle Eastern and Central South Asian ethnic components.

Admixture analysis and Y-chromosome haplogroup studies from Central/South Asian populations; in present-day Iran; showed consistent patterns with populations of the parts of the Greater Middle East (GME) including the Arabian Peninsula, revealing past movements and demographic events that contributed to the contemporary genetic landscape (Regueiro et al., 2006). Moreover, there were human migration from East African and increases in recent history through the trafficking of slaves into the Peninsula through several coastal routes including the trade route of Oman (Tadmouri et al., 1987). Intermixing has contributed to “genetic sharing” and hence the diversity of the local community (Tadmouri et al., 1987). The Levantine corridor and the Horn of Africa acted as two conduits that connected Africa and Eurasia, respectively (Luis et al., 2004). Furthermore, results from principal component analysis (PCA) of African populations (Cavalli-Sforza et al., 1993) and Y-chromosome data from Egyptian samples revealed genetic continuity between the people of the Nile River Valley and those of the Arabian Peninsula (Manni et al., 2002). People from Baluchistan in Central/South Asia (currently Iran, Afghanistan, and Pakistan) also moved freely in and out of the UAE and formed some very isolated communities with a high level of consanguineous marriages (Tadmouri et al., 1987) (see also Supplementary Figure S1).

All these events have left traces in the genetic structure of the contemporary population of the UAE. Nonetheless, current influx of foreign experts and laborers continue to contribute to shaping the unique composition of the population of the UAE.

Genotype arrays provide an inexpensive way to collect data from a large pool of samples, but are afflicted with preconceived knowledge about variant loci, and are not necessarily the most informative biomarkers for different world populations. In contrast, the use of WGS to study the genetic structure of a population, despite being more expensive, provides in-depth knowledge about archetypical genomes including novel variants.

This study is part of the 1,000 Arab genomes project (Al-Ali et al., 2018), intending to gain insights on the genomic variations by next generation sequencing (NGS) of the whole genomes of four UAE nationals. These subjects represent the different subpopulations that exist within and constitute the historical ethnic admixture that is found within the current UAE population. The selection of the four suitable subpopulation representatives has been informed by a preceding comprehensive population structure analysis (Tay et al., 2020) based on their subpopulation centrality and admixture. The novel strategies described in the paper have been used to gain a comprehensive overview of the UAE’s genetic landscape. The allele frequencies (AFs) from the microarray driven study were also used to characterize a substantial number of variants found in the four full genomes, thus providing an accurate means to glean variant rarity with respect to a hitherto understudied population. This, in turn, facilitated the understanding of rare and common genetic diseases found in the local UAE population.

The novel (short and structural) variants and the specific allele frequency calculated for the UAE resulting from this study fill a large gap in public human genomics databases. This study has also provided a starting point for designing a UAE reference panel which will lead to substantial improvements in medical applications that are expected to contribute to improvement in the local health care system.

This study also highlights the importance of understanding the genetic diversity of a population when identifying risk alleles and targeting genes that will lead to the development of novel therapeutic approaches given that the common variations among different populations could be very different form variants influencing disease risk and drug response (Lu et al., 2014).

## Materials and Methods

### Sampling and Sequencing

The four subjects UAE S011, UAE S012, UAE S013, and UAE S014, provided their written informed consent. The consent has been previously approved by the Institutional Ethics Committee of Mafraq Hospital in Abu Dhabi. The inclusion criteria of the study were: Emirati citizens, aged at least 18 years old, are capable to understand their contribution to the study, and have the ability to provide informed consent.

For the selection of the four samples, a method combining PCA and supervised admixture analysis described in Tay et al. (2020) was used. The admixture ratios were calculated and used to decide on the suitability of each sample to serve as representatives of the different subpopulations of the UAE for WGS. The samples’ physical characteristics are provided in Supplementary Table S2.

Saliva sample collection, DNA extraction and library preparation for the four subjects were performed using methods that support whole genome sequencing on an Illumina platform. The protocols were described in AlSafar et al. (2019).

### Alignment of Reads From Whole Genome Sequencing

Fastqc v0.11.5 (Andrews, 2010) and Trimmomatic (Bolger et al., 2014) tools were used for quality control (QC) and trimming of the raw reads -Trimmomatic PE -threads 30 -phred33 ILLUMINACLIP:TruSeq3-PE.fa:2:30:10 SLIDINGWINDOW:4:20 MINLEN:50 – which resulted in high quality reads for all four samples. The reads were mapped to the (hg19) version of the human genome reference sequence (Lander et al., 2001; Fujita et al., 2010) using the alignment “bwa mem” command from the BWA v0.7.12 (Li and Durbin, 2010) tool with the -M parameter to flag shorter split hits as secondary. The Qualimap software version 2.2.1 (Okonechnikov et al., 2016), was then used (with default parameters) to test the alignment results and coverage calculation.

For the SNPs and insertion/deletion (indels) discovery, Picard v2.9.4 (Liu X. et al., 2013) and Genome Analysis Toolkit (GATK) v3.7 (McKenna et al., 2010) (using best practice default values) were used for the processing of the aligned files (BAM) before the actual variant calling.

Variants were identified using the GATK HaplotypeCaller tool. The output was a Variant Call Format (VCF) (Danecek et al., 2011) file of variants classified into SNPs and indels. The Variant Quality Score Recalibration (VQSR) was used and filtration steps according to the GATK best practice recommended default values (tranche 99.0) (Van der Auwera et al., 2013; Carson et al., 2014) were performed. Classes of polymorphisms; SNPs and indels; were assessed and scored based on a standard Gaussian mixture model while using highly validated variant resources.

### SNPs and Indels Annotation

After filtering out all variants that did not pass GATK QC, the high-quality called variants from the four samples have been annotated using the genomic annotation tool SnpEff version 3.4 (Cingolani et al., 2012) which uses predictive algorithms to identify the functional effect of a variant in the genome. We furthermore used a comprehensive set of annotation databases, including dbNSFP (Liu X. M. et al., 2013), a database that compiles prediction scores from a wide range of annotations prediction tools. Furthermore, using AFs from the world and regional populations, we annotated the sequences with the genome aggregation database (gnomAD), the GME Variome project (Scott et al., 2016) and the UAE specific AFs – that were calculated from the genotyped data from 1,000 Emiratis. Both classes of variants (SNPs and indels) were further categorized into known and novel. The latter related to variants that were not reported in dbSNP versions 138 and 151 (Sherry et al., 2001). ClinVar (Landrum et al., 2014) was used to determine the clinical significance, disease associations, and linked phenotypes of the variants that were discovered. VCF-Miner (Hart et al., 2015), a graphical user interface was used for management and manipulation of the information encoded in the VCF files.

### Structural Variants Identification

The structural variants (SVs) for the four samples were generated using Pindel (Ye et al., 2009) and Breakdancer (Chen et al., 2009). Only SVs identified by both tools were considered. The result was then compared to the Structural Variants database (DGV) (MacDonald et al., 2013) to identify whether the UAE SVs were novel or known. The tool AnnotSV (Geoffroy et al., 2018), an integrated tool for structural variations annotation was used to annotate the identified SVs and the most prevalent medical implications from the Deciphering Developmental Disorders (DDD) of the respective SVs was reported.

### Estimating Ancestry to the Paternal and Maternal Linage of the Four WGS Haplogroups

Haplogroups for the Y-chromosome of the male participants (UAE S011, UAE S012, and UAE S014) have been assigned using yHaplo (Poznik, 2016). For mitochondrial (mt) haplogroup analyses, the mtDNA sequences from the BAM files from mapping the WGS of the four samples to the reference genome hg19, were lifted over to the revised Cambridge Reference Sequence (rCRS) (Andrews et al., 1999). The HaploGrep tool (Weissensteiner et al., 2016) was then used to determine the mtDNA haplogroups for the four samples.

### Genetic Ancestry and Mapping Against the World Populations

To define the genetic ancestry of the UAE population, a cohort of 1,000 UAE citizens were genotyped using the Illumina Omni 5 Exome bead chip which contains 4.6 million SNPs. After QC, merging with the Human Genome Diversity Project [HGDP] (2018) (Cann et al., 2002) data set (1,043 samples and 660,918 markers) yielded 235,478 SNPs as described in AlSafar et al. (2019) (AlSafar et al., 2019) and Tay et al. (2020). PCA was run using the PLINK’s pca command and the genetic ancestry for the four samples has been estimated using the ADMIXTURE tool (Alexander et al., 2009). The phylogenetic mapping of inter-genome distances between the genomes of the four samples and genomes from HGDP world populations have been performed using VCFtools (Danecek et al., 2011), PLINK (Purcell et al., 2007), BioPython’s Phylo modules and iToL2 (Letunic and Bork, 2011) as described in detail in AlSafar et al. (2019).

### Validation of the Variants Called for the Four Samples

Concordance between the Illumina Omni 5 Exome bead chip genotyped loci and the variants called for each of the four samples was calculated as a measure of validation for the called variants. The VCF files generated by the variant calling for UAE S011, UAE S012, UAE S013, and UAE S014 was converted to PLINK’s ped/map file format using vcftools (Danecek et al., 2011) resulting in 866,712 loci for each sample. A custom Python script was used to filter: missing values, Indels, multiallelic loci, and strand confusion before performing the final comparison between the two sets.

### Annotation With Local Allele Frequencies From Genotyping Data

The Emirati specific AFs are calculated from 1,000 Emirati samples for 2.3 million loci. In addition to the conventional annotation steps (see section SNPs and Indels Annotation), we used the resulting custom database in VCF format to additionally annotate the four genomes with the UAE AFs. The workflow is depicted in Supplementary Figure S3. We further filtered variants using the Variation Association Tools (VAT) (Wang et al., 2014).

The scripts that were used to generate the UAE allele frequency calculation from array data, structural variance (merging and identification of novel and known), annotation with allele frequency from (UAE, gnomAD, and GME), *Z*-score, concordance, PCA, ADMIXTURE are available at GitHub under: https://github.com/henschellab/populapy.

## Results

### Selection of Population Representatives for WGS and Their Admixture

The PCA and supervised admixture analysis method described in Tay et al. (2020) showed that the UAE population was weakly stratified and followed a gradient continuum admixture of mainly Middle Eastern, Central/South Asian, and Sub-Saharan African components.

The results of the study guided our choice of four samples (UAE S011, UAE S012, UAE S013, and UAE S014) from UAE nationals which according to their admixture ratios and subpopulation centrality, could serve as representatives of the different subpopulation of the UAE for WGS.

**FIGURE 1:**
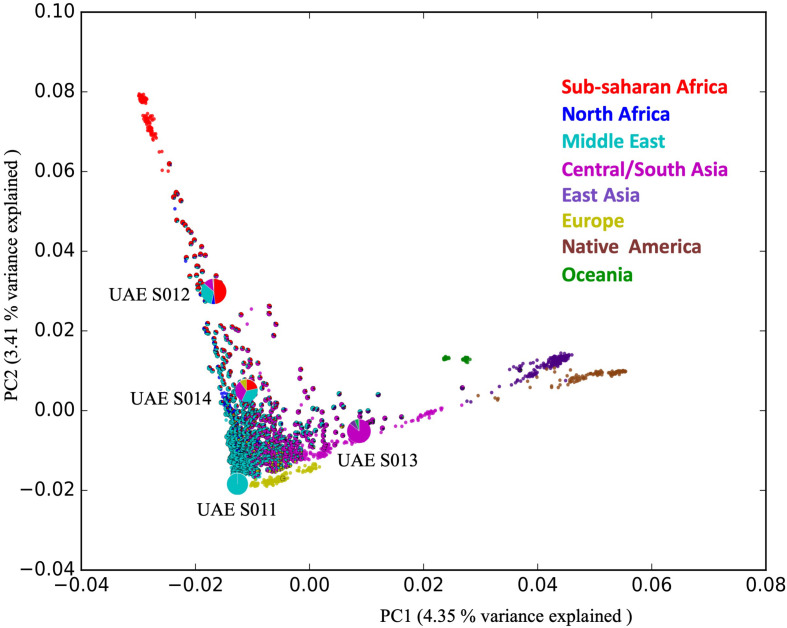
The positions of the four samples selected for WGS in the admixture PCA. The figure shows 1,000 UAE samples (shown as admixture pie charts) contextualized against 1,043 samples from different world populations taken from the Human Genome Diversity Project (HGDP), shown in non-outlined plain circles. The admixture ratio of the four distinct individuals (depicted by the large pie charts) representing the diversity within the population of the UAE.

The admixture distribution depicted by pie-charts and their position in the PCA plot illustrating the position of these four individuals in the context of the HGDP that includes populations from Africa, Central/South Asia, Eastern Asia, Native America, Europe, and Oceania is shown in Figure 1.

The analysis shows that the four samples are admixed according to the following ratios: UAE S011 (99.9% Middle Eastern), UAE S012 (48.3% Sub-Sahara African, 4.9% North African, 32.9% Middle Eastern, 12% Central/South Asian), UAE S013 (85% Central/South Asian, 7.9% East Asian, 5.5% Oceanian) and UAE S014 (21% Sub-Saharan African, 35% Middle Eastern, 10% European) as shown in Figure 1 (see Supplementary Table S1).

### Information on Alignment Statistics

The total number of the generated reads that passed QC ranged between (813,964,756 for UAE S011 and 613,562,075 for UAE S014). An average of 96% of the reads were mapped to the reference genome (hg19) (Lander et al., 2001; Fujita et al., 2010) with an average coverage of 33X (Supplementary Table S3).

### Observed Single Nucleotide Polymorphisms and indels

The four samples had an average of 4,427,749 identified variants per sample (with the highest being UAE S012 with 4,798,806). Of those variants, an average of 80% was found to be SNPs, and 20% indels (Supplementary Table S4). Variants were further characterized as “known” and “novel” with averages of 97 and 2.75%, respectively, depending on their presence or absence in dbSNP 151 and 138.

### Variants Validation Using Het/Hom, TI/TV Ratios and Concordance

The percentages of heterozygous (Het) and homozygous (Hom) variants ranged between 65.4 to 57.4% Het and 42.6 to 34.6% Hom (Supplementary Table S5).

The ratio of Het:Hom was generally expected to be around 2:1 (mainly for Africans) and the ratio drops lower for cases including highly consanguineous and less admixed populations (Wang et al., 2015). This was the case with some individuals in the UAE (see Figure 1). It was interesting to note that Het:Hom ratio of the two individuals with the greater degree of admixture; including the African component; tended to approach the 2:1 expected ratio (1.9:1 for UAE S012 and 1.7:1 for UAE S014) and these figures were inconsistent with this generalization (Wang et al., 2015). However, the ratios in the two individuals with one dominant ethnic background (1.3:1 for the mainly Middle Eastern UAE S011sample and 1.5:1 for the mainly Central South Asian UAE S013 sample) appeared to deviate from expectations suggesting high homozygosity of this admixture.

The genome-wide transition (TI)/transversion (TV) ratios in the four samples were summarized in Supplementary Table S6. All samples scored a TI/TV ratio of ∼2.05 for total and known variants consistent with previous reports (Bentley et al., 2008; Pelak et al., 2010).

Furthermore, the classifications of the known and novel variants based on their effect impact on genes (i.e., how deleterious the variant is on genes), the functional class of the variant (nonsense, missense, or silent) and the effect of the variants by type (e.g., stop gain, frameshift, etc.) and by region (e.g., downstream, exon, etc.) as per SnpEff annotation were shown in Supplementary Tables S7–S9.

Furthermore, the WGS results were compared to the genotyping data of the four sequenced individuals acquired from the Illumina Omni 5 bead chip technology using concordance calculations. After QC and filtering (see section “Validation of the Variants Called for the Four Samples”) the final comparison was based on 245,697 SNPs for (UAE S011), 266,208 SNPs for (UAE S012), 245,308 SNPs for (UAE S013), and 259,124 SNPs for (UAE S014), the intersection of the filtered SNP loci and the single nucleotide variant calls from NGS yielded concordance percentages of >99% for the four samples as listed in Supplementary Table S10.

### Estimating Ancestry Using the Y-Chromosome and Mitochondrial Haplogroups of the Participants

The lineage markers presented in the four sequenced individuals were found to be: the Y haplogroup was identified for three male samples UAE S011, UAE S012, and UAE S014 using AMY-tree and yHaplo. Both tools determined that the three male individuals belonged to the Y-haplogroups: J1 (UAE S011), E1 (UAE S012), and J2 (UAE S014), respectively.

The predominantly Middle Eastern subject (UAE S011) and the equally admixed sample (UAE S014) shared the same H2 mtDNA haplogroup mainly originating from West Asia. They also both shared the J Y-haplogroup with subclades J1 (UAE S011) which was found in the greatest concentration in the Arabian Peninsula (Abu-Amero et al., 2009) and J2 (UAE S014) which is West Asian in origin (Rodriguez-Flores et al., 2016). The UAE S012 sample was assigned the E1 Y-haplogroup and the L0 mtDNA haplogroup both found mainly in Africa (Harich et al., 2010) consistent with the high African ratio of its admixture.

Sample UAE S013, being a female, we only defined its mtDNA haplogroup (M18) which was a dominant South Asian haplogroup (Rodriguez-Flores et al., 2016) in agreement with the 85% central/South Asian ratio of the sample’s admixture.

### Phylogenetic Contextualizing of the Four Samples Against World Populations

**FIGURE 2:**
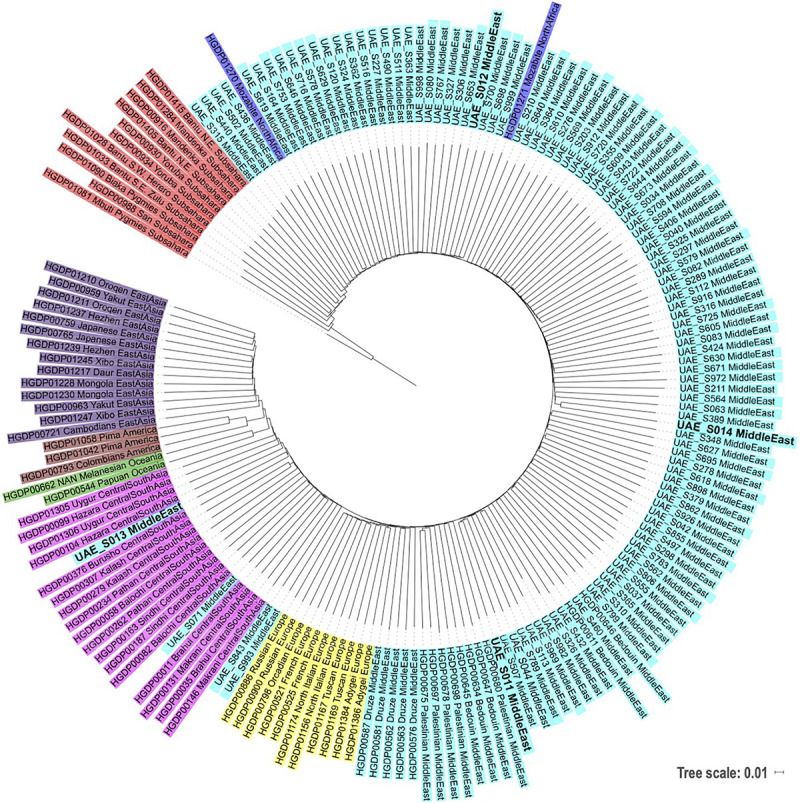
Phylogenetic tree based on neighbor-joining and identity by state distances contextualizing local samples against samples from various world populations from HGDP (following the same populations coloring scheme as in Figure 1). The subjects of this study are displayed in bold.

A phylogenetic tree was reconstructed using the neighbor-joining method containing the four subjects (in bold) as shown in Figure 2. The UAE S011 and UAE S014 Emirati samples grouped well within the Middle Eastern Arabs. Similarly, UAE S012 fell within the Middle Eastern group but in closer proximity to the Sub-Saharan African group. UAE S013 was situated centrally in the Central/South Asian group. These phylogenetic locations were in agreement with their admixture and Y/mtDNA haplogroups results.

### Observed Structural Variants and Its Annotations

The called SV totaled 15,677 to 20,339 of which only around 13.5% were novel. Supplementary Table S12 summarizes the numbers of SVs identified and groups them into known and novel based on their availability in the DGV database. The SVs were further grouped by the three SV types; deletion, inversion and insertions; and listed in Supplementary Table S13.

The SVs were annotated with their respective disease association from the DDD study (Firth et al., 2011) and the results for the four samples in the form of the most frequently annotated disease (only confirmed as per DDD status) were reported in Supplementary Tables S18–S21, respectively.

It was interesting to note that the four samples share a high annotation frequency (>10) for the Wolcott–Rallison syndrome, a rare autosomal recessive disease, characterized by neonatal or early-onset non-autoimmune insulin-requiring diabetes associated with skeletal dysplasia and growth retardation (Julier and Nicolino, 2010).

### Comparison of the Called Variants of the Four Samples

After comparing the results of the variants that were identified for the four individuals, it was found that the four samples shared 1,399,178 (30–34%) variants, and each had distinct variants as follows: 1,085,524 (UAE S011), 1,228,559 (UAE S012), 791,072 (UAE S013), and 906,818 (UAE S014) resulting in a percentage of between 19 and −27 percent of variants compared to their respective total called variants (see Supplementary Figure S2).

The number of individual-specific variants for such a sampling size was comparable to inter-population sampling, as with the example documented in Almal et al. (2018). An overlap of 37.88% and a distinct variant percentage of 14.61% were found when comparing the genome of a Gujarati Indian individual to four different genomes from Asia; Pathan-Pakistani, Chinese, Japanese, and Korean.

This phenomenon can be attributed to the fact that the population structure analysis (see Figure 1) evidences a vast extent of heterogeneity, hence showing a previously unappreciated diversity.

### Variants Associated With Specific Diseases

**FIGURE 3:**
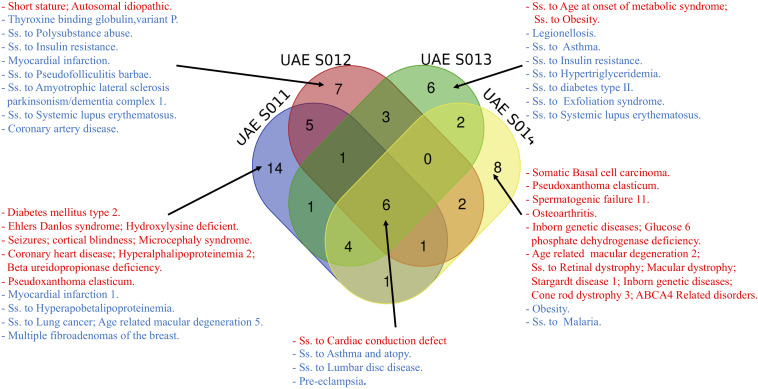
The different disease names (Ss is short for Susceptibility) from common and distinct variants among the four samples identified as pathogenic (red)/risk factor (blue) according to ClinVar.

It is important to delineate the associations between genotypes and disease for personal genomes by relating the variants with potential susceptibility to an extensive list of disorders. Figure 3 illustrates the different common and distinct disease variants among the four samples that were identified as pathogenic (red)/risk factor (blue) according to ClinVar. The common diseases include susceptibility to cardiac conduction defect, asthma and atopy, lumbar disc disease and pre-eclampsia, with all with high values of UAE specific AFs falling between 0.4783 and 0.8343, making them common variants for the UAE population. Although, numerous disease associations were identified as unique to each of the four genomes, most were not those which are highly prevalent in the UAE, such as Type 2 Diabetes Mellitus (T2D), cardiovascular disease, asthma, and cancer (Loney et al., 2013; see Supplementary Tables S14–S17).

Additionally, two interesting examples in samples UAE S011 and UAE S014 were identified, where a *Z*-score > 4 showed a significant difference in the UAE specific AFs of the two pathogenic variants. Firstly, the rs58073789 variant found in UAE S011 was related to Pseudoxanthoma Elasticum. It carried a UAE allele frequency as high as 4.96%, considerably higher compared to other world population frequencies, e.g., Europeans have 1.01% allele frequency (see Supplementary Table S14).

Secondly, the variant (rs1800553) from UAE S014 was described by ClinVar as “age-related macular degeneration 2, susceptibility to retinal dystrophy, inborn genetic diseases, cone rod dystrophy 3, ABCA4 Related Disorders” and has a UAE allele frequency as high as 1.9% (see Supplementary Table S17).

### Variant Prioritization With UAE Specific Allele Frequencies for the Four Samples

A method was described for annotating the variants of four new genomes, not only against the list of gnomAD AFs but as much as possible (between 686,508 and 723,586 variants) with AFs from comprehensive local genotype arrays, thus establishing a technique for up- and down-prioritizing variants (Supplementary Figure S3). The rationale was that variants with comparatively high local AFs (high positive *Z*-score) were unlikely to be disease causing as they show no sign of negative selection. Conversely, of particular interest were variants that were recognized to be rare in the country (negative *Z*-score), as they possibly indicate a less permissive mutation. A substantial number of variants were identified for each genome that has significantly different AFs when compared to gnomAD populations. Between 2,498 and 5,254 called and array-genotyped variants per genome had *Z*-scores greater than 4, i.e., those variants have significantly elevated AFs in the UAE. On the other hand, between 885 and 964 variants per genome were relatively rare in the country and should receive greater scrutiny.

Of those variants annotated with indications for (potential) pathogenicity or deleteriousness, rare and common variants were identified for the local population. Analysis revealed between 167–280 protein-altering variants (out of approximately 5,300) that were common. These common protein alterations might account for benign traits. The summary of these findings was listed in Table 1.

**TABLE 1:** Variants with significantly different local allele frequencies (AFs) annotated with pathogenicity or (potential) deleteriousness, rare and common variants have then been identified with respect to the local population using the *Z*-score.

Sample ID	UAE AF			ClinVar non-benign			ClinVar pathogenic/risk factor		
	All *Z*	*Z* ≤ −4	*Z* ≥ 4	All *Z*	*Z* ≤ −1.96	*Z* ≥ 1.96	All *Z*	*Z* ≤ −1.96	*Z* ≥ 1.96
UAE S011	686,508	885	5,254	205	1	29	41	0	3
UAE S012	723,586	964	2,750	198	1	11	38	0	0
UAE S013	687,615	928	2,498	152	1	6	29	0	0
S014	715,338	951	2,938	175	0	8	35	0	0

**Sample ID**	**SIFT deleterious**			**SIFT/polyphen deleterious**			**SnpEff protein altering**		
	**All *Z***	***Z* ≤ 1.96**	***Z* ≥ 1.96**	**All *Z***	***Z* ≤ −1.96**	***Z* ≥ 1.96**	**All *Z***	***Z* ≤ −1.96**	***Z* ≥ 1.96**

UAE S011	784	11	30	882	11	37	5,292	51	280
UAE S012	827	12	18	936	12	23	5,529	53	167
UAE S013	782	11	16	879	11	20	5,194	51	175
UAE S014	820	14	19	916	14	26	5,418	50	184

Note that the strategy that we have outlined is limited by the incomplete nature of deriving AFs from genotype arrays, but the chosen chips were already capturing millions of the most variable loci of the human genome and thus constituted a cost-effective option to approximate a substantial subset of the UAE variome.

## Discussion

This study is particularly important because it has provided genome sequences from an admixed and underrepresented population. Such genomes are essential to fill the gap in the global human genomics efforts. It is important to generate more ethnic-specific reference genomes to improve the balance of information to improve the identification of genetic variations that are population-specific (Li et al., 2010; Lu and Xu, 2013; Popejoy and Fullerton, 2016).

The population analysis in Tay et al. (2020) described the genetic landscape of the UAE population using an informative tool and strategy for selecting subpopulation representatives in terms of admixture and subpopulation centrality. In contrast to the first two Emirati genomes in AlSafar et al. (2019), where they were chosen from samples of similar ethnic admixture for the sake of comparability, the genomes of this study are a more adequate representation of the diversity and different admixture in the UAE population, a point that needs to be considered in future anthropological and medical applications.

These four genomes have enabled us to gain insights into common and subpopulation specific disease-associated variants. An analysis of variant overlap between the four representatives of the ethnic group in the UAE revealed a high number of individual variants, comparable to interpopulation samples. While this is also a consequence of the deliberate sample selection guided by a co-conducted large scale population structure analysis, the overall heterogeneity that was observed in the UAE exceeded those of many world populations (Tay et al., 2020) and thus further motivates the establishment of reference genomes for subpopulations.

Another notable result is the use of the UAE specific AFs that were calculated from the genotype arrays of 1,000 UAE random samples to perform variant prioritization in available and future genomes from the UAE. Although WGS/WES derived AFs like ExAC and gnomAD (Lek et al., 2016) are more comprehensive with respect to the number of loci, a substantial part of locally observed and clinically relevant variants can be annotated with local AFs. As many loci in the UAE population have significantly different AFs in contrast to all other world populations of gnomAD, they facilitate the identification of rare and common variants more accurately.

Combining the breadth from array genotyping and the depth from high coverage NGS of whole genomes, the data collected in this study has provided a rich base for future genomic studies in the country and in the region, which is notoriously understudied. On the one hand, the four genomes will work as a more suitable reference for the next “n + 1” local sequencing effort, since it is very likely that at least one of the genomes is a more adequate match than the conventional reference genomes. Albeit the presented genomes are likely to contain minor AFs with respect to the local population, they still constitute a better overall match and warrant a more precise variant calling process than references like hg19 or GRCh38, that have been generated without input from the region. However, even in the small population at hand, due to strong diversity introduced from major contributions of continental world populations, the choice of a reference is not trivial. Here, it is evident that affordable genotyping will help establish a comprehensive population overview, which in turn provides guidance in choosing adequate representatives in terms of their centrality in the population and their admixture. It is thus possible to use genotype array data and NGS as complementary sources of data for anthropological studies as well as personalized medicine.

Through the use of these four individual genomes, we are able to ascertain ethnic affiliation through the analysis of the Y-chromosome and mtDNA haplogroups in an attempt to develop paternal and maternal ancestors’ migratory patterns for the particular subgroups as well as developing health profiles of the different individuals. The results of the analysis have been in line with our prediction using historical records. Furthermore, the current analysis presents a high-quality WGS effort in the UAE including their clinical and functional annotations and identification of novel variants that benefited from filtering using the UAE specific AFs.

The underrepresentation of the UAE in global genetic databases (Popejoy and Fullerton, 2016) makes the implementation of precision medicine tools and therapies in the UAE to be challenging when using genome references and AFs from other populations.

This study has highlighted the importance of analyzing interpopulation genetic diversity in the admixed population of the UAE for providing more adequate risk alleles for genetic diseases in similar populations. This is especially important when analyzing and interpreting their results for investigating genomic medical applications such as personalized medicine and a more accurate identification of risk alleles in these populations.

Furthermore, limited analysis of novel SV association to disease indicated a link among the 4 samples to the Wolcott–Rallison syndrome a disease characterized by neonatal/early-onset non-autoimmune insulin-requiring diabetes according to (DDD). Further investigation of the SVs disease associations is needed but is outside the scope of this study.

## Data Availability Statement

The data is available at the European Genome Archive (EGA) under the accession number “EGAS00001004389.”

## Ethics Statement

The studies involving human participants were reviewed and approved by the Mafraq Hospital in Abu Dhabi, United Arab Emirates (UAE). The patients/participants provided their written informed consent to participate in this study.

## Author Contributions

HA obtained the funding for this study. HA and GT designed the study. GT, GD, and AH analyzed the data and prepared the manuscript. GT, HA, GD, and AH reviewed and edited the manuscript and contributed to the discussion. All authors contributed to the article and approved the submitted version.

## Conflict of Interest

The authors declare that the research was conducted in the absence of any commercial or financial relationships that could be construed as a potential conflict of interest.
