# Supplementary material for: Whole Genome Sequencing of Four Representatives From the Admixed Population of the United Arab Emirates
Source: Front Genet. 2020 Jul 9;11:681. doi: 10.3389/fgene.2020.00681 (PMC7367215; doi:10.3389/fgene.2020.00681)
Supplement: Supplementary file 1 [file Data_Sheet_1.docx]

Supplementary Material

1. Supplementary Figures


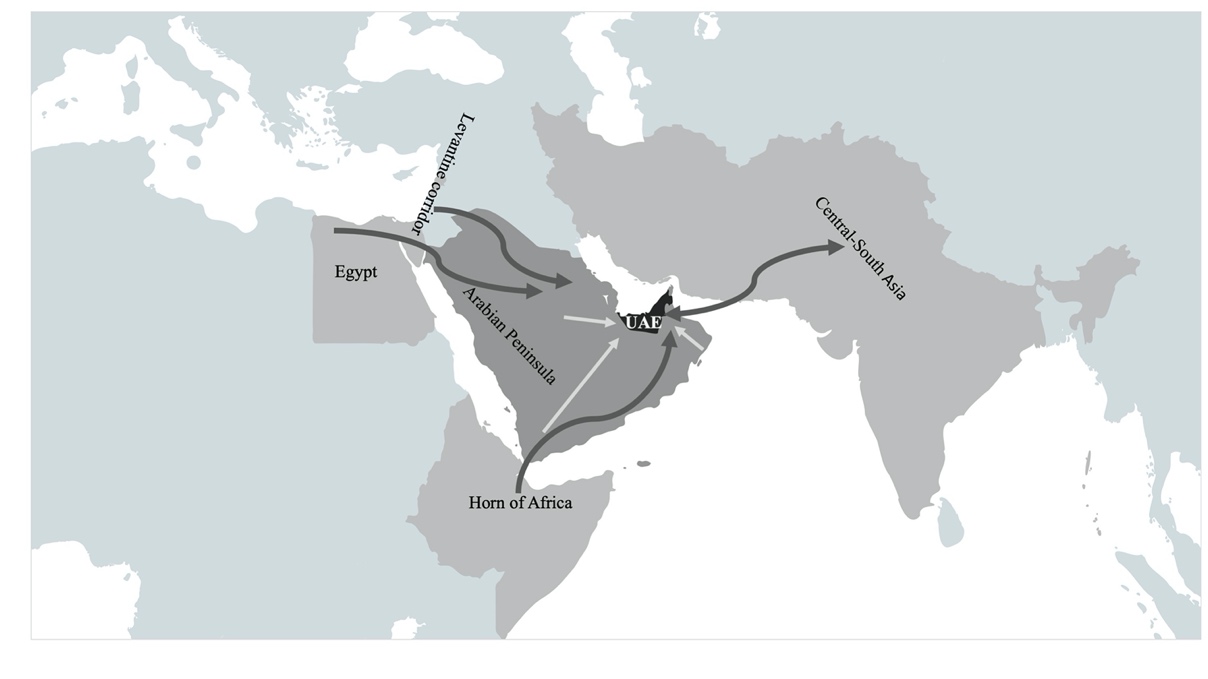


Figure S1: A number of ancient and recent migration routes into the Arabian Peninsula have influenced the genetic make-up of the UAE population. East African populations moved into the Arabian Peninsula through the Southern trade routes from the Horn Africa and into the UAE. The Levantine corridor was the migration route that connected Europe to Asia, passing through the Arabian Peninsula including the UAE. Commonality of some genetic markers between populations that inhabited the Nile River Valley in Egypt and those of the Arabian Peninsula further suggest a migration route from North Africa. Populations in Central-South Asia (now Iran, Afghanistan and Pakistan) have moved in and out of the UAE throughout history. These migration events have combined with admixing between neighboring countries (light arrows) within the Arabian Peninsula to contribute to the diversity of the contemporary population of present-day UAE.


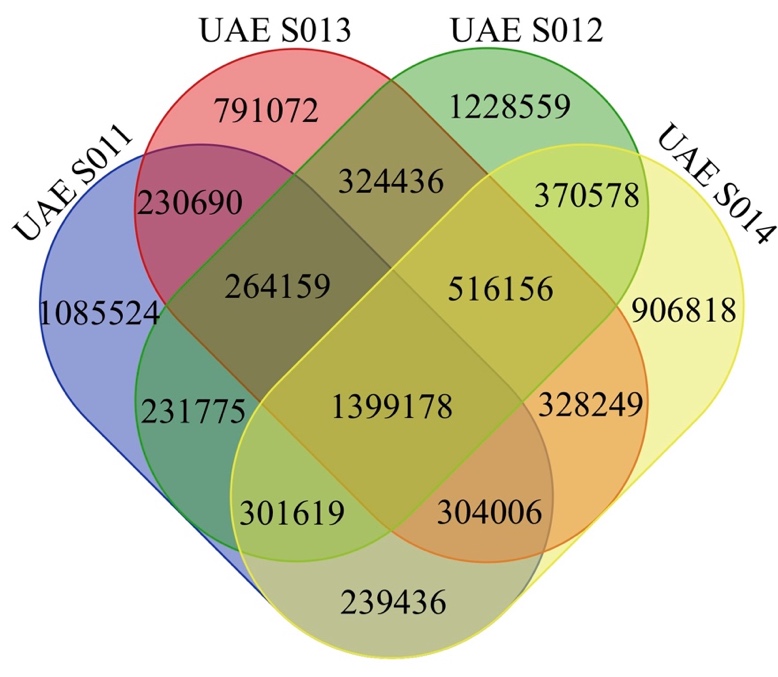


Figure S2: Comparisons between the called variants of the four UAE samples. The overlaps show that common variants between pairwise comparisons of the 4 samples ranged from 30% to 34%. Nineteen to 27 percent of the variants were unique.


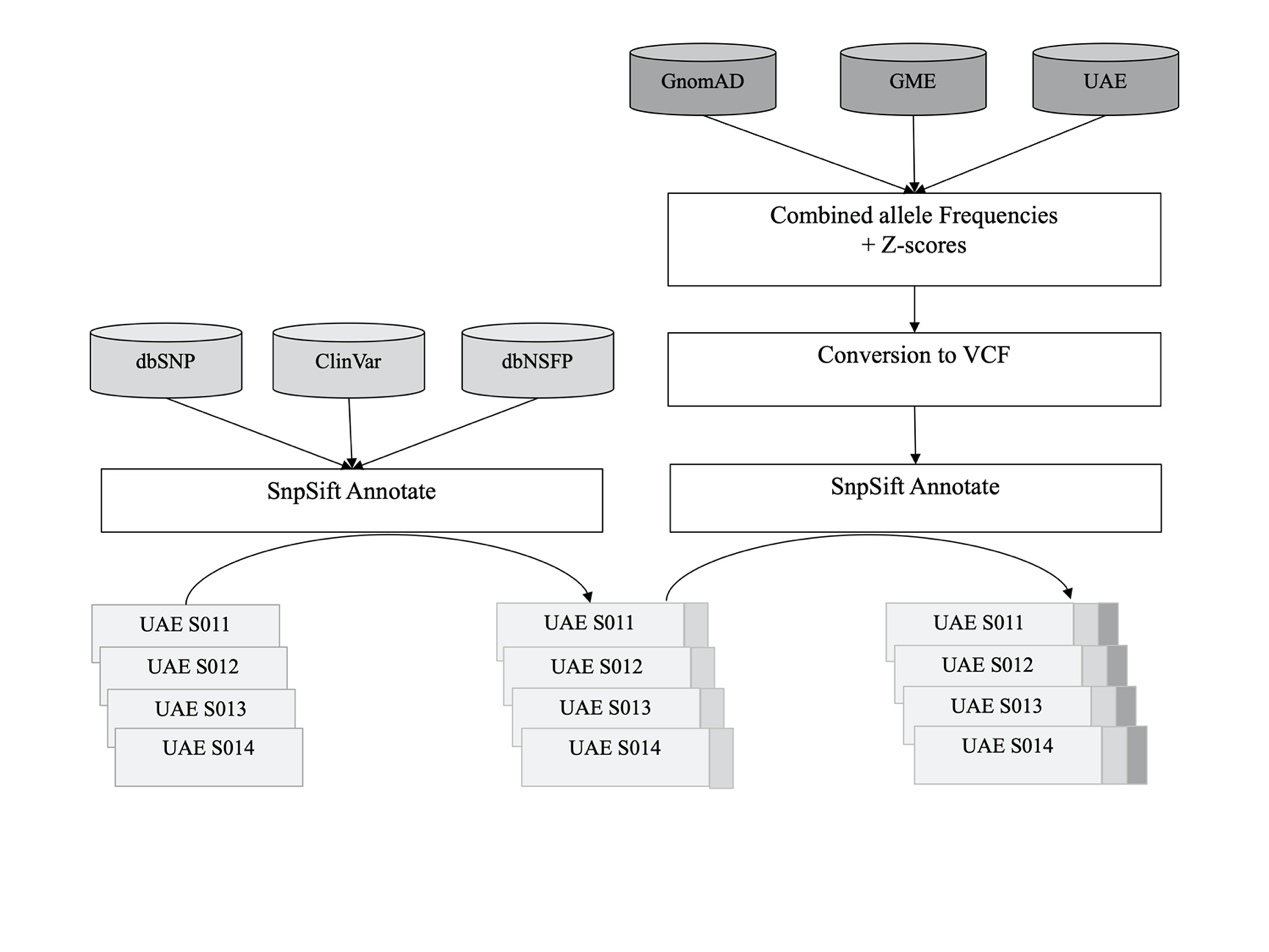


Figure S3: The annotation workflow for the 4 UAE genomes included comparisons with customized and conventional annotation databases. The allele frequencies from 3 custom datasets were used, namely those constructed from (1) the population from GnomAD, (2) a GME populations (3) the 1,000 UAE genome population. These variants were subsequently annotated against information from dbSNP, ClinVar and dbNSFP.

# Supplementary Tables

Table S1: Admixture ratios for the 4 samples studied based on the eight HGPD populations. The ratios were obtained by using the ADMIXTURE tool.

Table S2: Characteristics of the four samples studied by WGS.

Table S3: Alignment statistics and genome coverage for the four participants.

Table S4: Variants for the four samples: called known and novel SNPs and insertions and/or deletions with reference to dbSNP build 151.

Table S5: The number of known homozygous and heterozygous variants in the fours samples studied with reference to dbSNP build 138 and 151.

Table S6: The TS/TV ratios of the variants called for the four samples. All samples scored a ratio of ~2, which is the expected value for a WGS.

Table S7: Functional classifications of the known and novel variants based on their SnpEFF genome annotation impact.

Table S8: Classification of the known and novel variants of the four samples based on their SnpEFF functional class.

Table S9: Summary of the known and novel variant of the four samples classified by type using SnpEFF within the different genomic locations.

Table S10: Concordance in SNP calls between the WGS and the genotyping experiment using Bead Chip array.

Table S11: Maternal and paternal lineage markers in the four samples studied.

Table S12: Total number of known and novel structural variants identified by taking the consensus of the Pindel and Breakdancer tools outputs and subsequent comparisons against DGV.

Table S13: The number of structural variants by type (deletion, inversion and insertion).

Table S14: A list showing variants of clinical significance of variants in UAE S011. The allele frequencies of these variants from the UAE 1,000 GWAS study and five gnomAD population groups (GME, EAS, ASJ, AFR and NFE) are also shown.

Table S15: Clinical significance, functional annotation and allele frequencies from the UAE and gnomAD of known variants in UAE S012.

Table S16: Clinical significance, functional annotation and allele frequencies from the UAE and gnomAD of known variants in UAE S013.

Table S17: Clinical significance, functional annotation and allele frequencies from the UAE and gnomAD of known variants in S014.

Table S18: Frequency of structural variants found in UAE S011 that are associated with Deciphering Developmental Disorders (DDD).

Table S19: Frequency of structural variants found in UAE S012 that are associated with Deciphering Developmental Disorders (DDD).

Table S20: Frequency of structural variants found in UAE S013 that are associated with Deciphering Developmental Disorders (DDD).

Table S21: Frequency of structural variants found in UAE S014 that are associated with Deciphering Developmental Disorders (DDD).
